# HSP90α plays an important role in piRNA biogenesis and retrotransposon repression in mouse

**DOI:** 10.1093/nar/gku881

**Published:** 2014-09-27

**Authors:** Tomoko Ichiyanagi, Kenji Ichiyanagi, Ayako Ogawa, Satomi Kuramochi-Miyagawa, Toru Nakano, Shinichiro Chuma, Hiroyuki Sasaki, Heiichiro Udono

**Affiliations:** 1 Department of Immunology, Okayama University Graduate School of Medicine, Dentistry, and Pharmaceutical Sciences, Kita-ku, Okayama 700-8558, Japan; 2 Division of Epigenomics and Development, Medical Institute of Bioregulation, Kyushu University, Higashi-ku, Fukuoka 812-8582, Japan; 3 Department of Pathology, Medical School and Graduate School of Frontier Biosciences, Osaka University, Suita, Osaka 565-0871, Japan; 4 CREST, Japan Science and Technology Agency (JST), Saitama 332-0012, Japan; 5 Department of Development and Differentiation, Institute for Frontier Medical Sciences, Kyoto University, Sakyo-ku, Kyoto 606-8507, Japan

## Abstract

HSP90, found in all kingdoms of life, is a major chaperone protein regulating many client proteins. We demonstrated that HSP90α, one of two paralogs duplicated in vertebrates, plays an important role in the biogenesis of fetal PIWI-interacting RNAs (piRNA), which act against the transposon activities, in mouse male germ cells. The knockout mutation of *Hsp90α* resulted in a large reduction in the expression of primary and secondary piRNAs and mislocalization of MIWI2, a PIWI homolog. Whereas the mutation in *Fkbp6* encoding a co-chaperone reduced piRNAs of 28–32 nucleotides in length, the *Hsp90α* mutation reduced piRNAs of 24–32 nucleotides, suggesting the presence of both FKBP6-dependent and -independent actions of HSP90α. Although DNA methylation and mRNA levels of L1 retrotransposon were largely unchanged in the *Hsp90α* mutant testes, the L1-encoded protein was increased, suggesting the presence of post-transcriptional regulation. This study revealed the specialized function of the HSP90α isofom in the piRNA biogenesis and repression of retrotransposons during the development of male germ cells in mammals.

## INTRODUCTION

Heat-shock protein 90 (HSP90), the most abundant protein in mammalian cells, is a chaperone that stabilizes the conformation of >200 client proteins in various physiological pathways, thereby maintaining cellular homeostasis (1,2). In mammals, there are two cytosolic HSP90 isoforms encoded by distinct genes, *Hsp90aa1* (HSP86; HSP90α) and *Hsp90ab1* (HSP84; HSP90β) sharing 86% similarity in amino acid sequences, as well as HSP90 family proteins localized in the mitochondria and endoplasmic reticulum. For simplicity, we refer to *Hsp90aa1* and *Hsp90ab1* as *Hsp90α* and *Hsp90β*, respectively. Although *Hsp90β* is ubiquitously expressed (constitutive type), *Hsp90α* expression is increased in response to various stresses (inducible type), and its expression is more tissue-specific at the steady state, being relatively higher in the testes and brain (3–5). Whether these HSP90 proteins have specific functions remains unclear.

Recently, plant and insect HSP90 proteins were implicated in the biogenesis of three major classes of small RNAs: small interfering RNA (siRNA), microRNA (miRNA) and PIWI-interacting RNA (piRNA). In insects and plants, the ATPase activities of HSP90 and HSP70 are indispensable for the formation of the pre-RNA-induced silencing complex (pre-RISC), in which double-stranded RNA precursors of siRNA and miRNA are loaded onto Argonaute proteins (6–8). In cultured human cells, however, chemical inhibition of HSP90 proteins does not affect miRNA expression, although Argonaute-2 is mislocalized (9). In animal gonads, PIWI-clade Argonaute proteins (Piwi, Aub and Ago3 in flies and MILI, MIWI and MIWI2 in mice) play a principal role in the generation of piRNAs, germline-specific small RNAs typically 24–33 nucleotides (nt) in length that counteract the transposon activities (10). In flies, Hsp90 has been implicated in piRNA production (11,12), and its co-chaperone, Hop, regulates Piwi phosphorylation and thus piRNA production (12). Moreover, silkworm Hsp90 participates in the loading of piRNA precursors onto Piwi (13). Another Hsp90 co-chaperone, Shutdown, is important for piRNA production in flies (14), and its mouse ortholog, FKBP6, has been proposed to facilitate the recycling of PIWI proteins in piRNA biogenesis (15). However, whether HSP90 plays a role in piRNA biogenesis in mice and other vertebrate species remains unknown. Studies on the role of the mouse HSP90 proteins in piRNA biogenesis have been hindered by the presence of two *Hsp90* genes in mice versus a single gene in insects. *Hsp90α* knockout (KO) mice are viable, presumably because of the presence of functional *Hsp90β*. However, we revealed that KO males are infertile due to a failure in spermatogenesis (16,17), suggesting that *Hsp90α* plays a role in spermatogenesis that cannot be replaced by *Hsp90β*. In this study, we investigated piRNA biogenesis in *Hsp90α* KO mice and revealed a specific function of HSP90α in the piRNA-based host-defense system against transposons in mice.

## MATERIALS AND METHODS

### Animals

The *Hsp90α* and *Mitopld* KO strains were described by Kajiwara *et al.* (17) and Watanabe *et al.* (18), respectively.

### Antibodies

Polyclonal antibodies to HSP86 (HSP90α) and HSP84 (HSP90β) were purchased from Thermo Scientific (PA3–013, RB-118). For immunostaining, polyclonal antibodies against MILI (PIWIL2), MIWI2 (PIWIL4) and WDR77 were purchased from Abcam. Anti-TDRD1 and TDRD9 polyclonal antibodies and anti-MIWI2 polyclonal antibody for immunoprecipitation were made by S. C. (Kyoto University) and S. K.-M. (Osaka University), respectively. Anti-L1 ORF polyclonal antibody and *Mael* KO testes lysate were generous gifts from Dr A. Bortvin (Carnegie Institute) (19). Anti-mono and dimethyl arginine monoclonal antibody (7E6) used in western blot analysis was obtained from Abcam.

### Oligonucleotides

The sequences of oligonucleotides used in this study are listed in Supplementary Table S1.

### Immunofluorescence detection of protein localization

E16.5–18.5 testes were embedded in OCT compound and snap-frozen in liquid nitrogen or isopentane cooled in liquid nitrogen. Cryosections were cut at 7–10-μm thickness and air-dried. The sections were fixed for 10 min in 4% PFA at 4°C, washed with PBS, permeabilized in PBS with 0.5% Triton X-100 for 30 min at room temperature, and blocked in PBS with 0.1% Triton X-100 and 1% BSA for 30 min at room temperature. Primary antibodies were incubated overnight at 4°C. Slides were washed and incubated with Alexa Fluor 488-labeled secondary antibody for 2–3 h at room temperature. The sections were mounted with SlowFade Gold Antifade Reagent with DAPI (Life Technologies) and observed by fluorescence microscopy.

### Small RNA sequencing analysis

Total RNA was extracted from wild-type (WT) and *Hsp90α* KO testes on E16.5 (20 testes for each condition) by Isogen (Toyobo, Japan) and used to create a small RNA library using the TruSeq Small RNA Library Preparation Kit (Illumina). The libraries were sequenced on MiSeq (Illumina) via 50-bp single-end sequencing. After clipping the adaptor sequence, sequence reads of more than 10 bp were mapped to cellular RNA and miRNA sequences in miRBase (20) by SeqMap (21). Unmapped sequences were then mapped to the mouse genome sequence, allowing no mismatch, and the mouse transposon sequences obtained from RepBase (22), allowing 2-bp mismatches. For piRNA cluster-derived RNAs, the sequences uniquely mapped to the fetal piRNA clusters (23) were analyzed.

The small RNA sequencing data were obtained from Gene Expression Omnibus (GEO) for *Fkbp6* KO and its WT control (GSE39203). These data and our published data for *Mili* KO, *Miwi2* KO and their WT control (GSE20327) were analyzed using the aforementioned reference sequences.

### Immunoprecipitation and western blotting

Three pairs of E16.5 testes were lysed in lysis buffer [20 mM HEPES pH 7.5, 150 mM NaCl, 2.5 mM MgCl_2_, 0.1% Nonidet P-40 (NP40), 1 mM DTT, protease inhibitor cocktail (Nacalai, Japan)] on ice using a Dounce homogenizer, and the lysate was cleared by centrifugation at 17,800 × g for 15 min at 4°C. The lysate was incubated with anti-MIWI2 antibody overnight at 4°C, following which Protein A/G PLUS-Agarose was added to collect the antibody and protein complexes. The agarose beads were washed four times with wash buffer (25 mM Tris-HCl pH 7.5, 150 mM NaCl, 2.5 mM MgCl_2_, 0.05% NP40 supplemented with 1 mM DTT, protease inhibitor cocktail). Immunoprecipitants were eluted in sodium dodecyl sulphate (SDS) sample buffer and run on an SDS-polyacrylamide gel electrophoresis gel. Western blotting was performed as described previously (24).

### Methylation-sensitive Southern blotting

The genomic DNAs of WT or *Hsp90α* KO testes at P14 (5 μg each) were digested by MspI (methylation insensitive) or HpaII (methylation sensitive) and run on a 0.8% agarose gel. DNAs were then transferred to a Hybond XL membrane (GE Healthcare), hybridized with a ^32^P-labeled L1 probe (25) at 42°C for 20 h and washed four times with wash buffer (2× SSC, 0.1% SDS) at 42°C. The radioactivities on the membrane were detected on a BAS2500 analyzer (Fujifilm, Japan).

### Northern blotting

Total RNA (10 μg each) was run on a 15% polyacrylamide gel, transferred to a Hybond XL membrane using a semi-dry method (100 mA, 2 h) and cross-linked by UV. The membrane was hybridized against radioactive probes at 40°C overnight and washed four times in wash buffer (2× SSC, 0.1% SDS). The L1 (piR-1831) and Intracisternal A particle (IAP) (piR-4868) probes used were described previously (25).

### Germ cell preparation and bisulfite sequencing

Germ cells were purified by fluorescence activated cell sorting. Prospermatogonia were isolated from P0 testes of mice carrying an Oct4-EGFP transgene. Spermatocytes were isolated from P17 testes as described previously (26). Genomic DNA was isolated by standard procedure and used for bisulfite sequencing as described previously (27). For each germ cell preparation, the cell purity was validated by confirming the percentages of DNA methylation in the Lit1 differentially methylated region, which is completely unmethylated in male germ cells and 50% methylated in somatic cells.

## RESULTS

### *Hsp90α* mutation affects the localization of MIWI2, a PIWI protein

**Figure 1.**
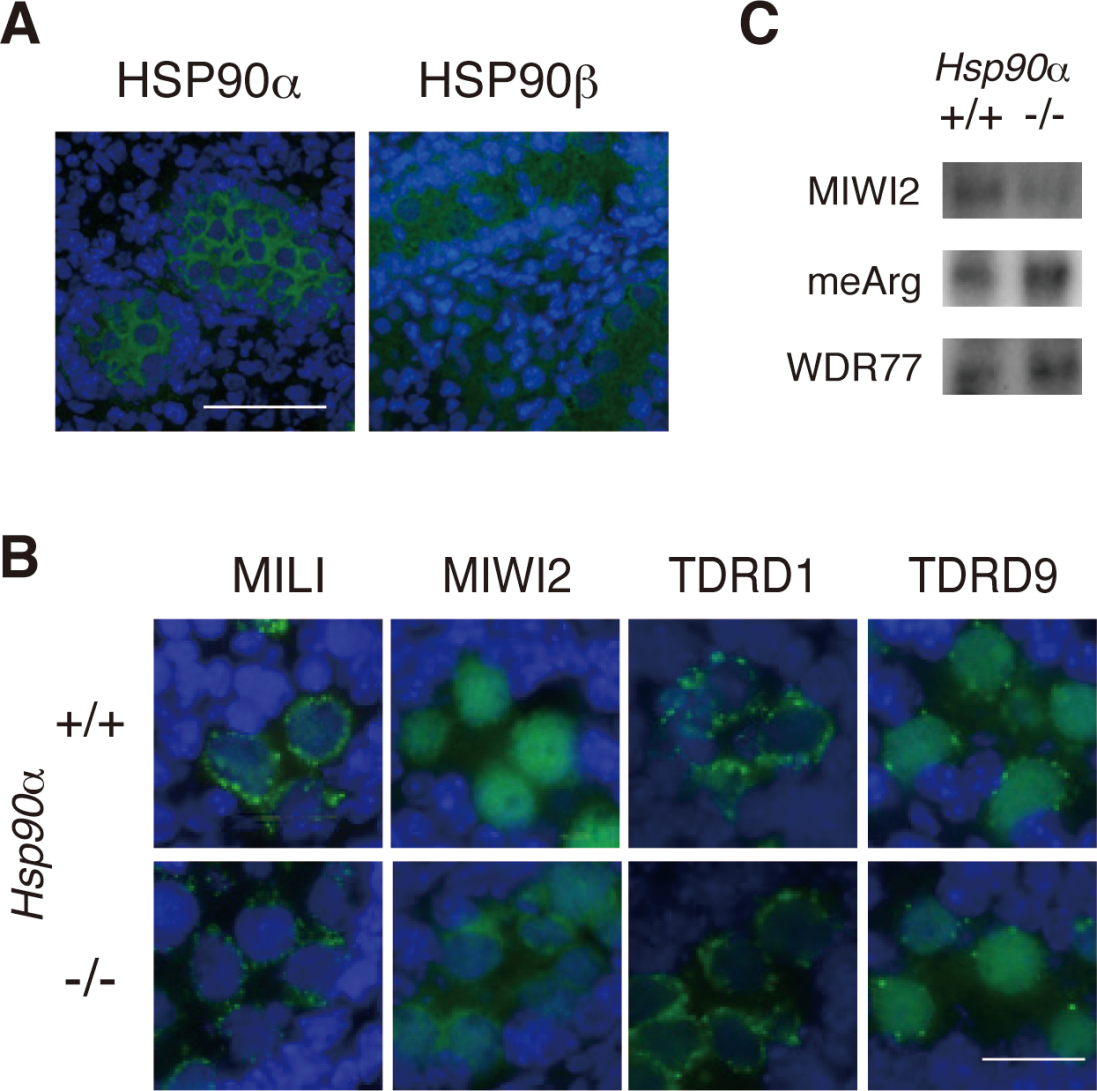
The *Hsp90α* mutation affected the localization but not the arginine methylation of MIWI2. (**A**) Immunofluorescence staining of HSP90α (green; left) or HSP90β (green; right) in WT E18.5 testes. Nuclei were counter-stained with DAPI (blue). (**B**) Localization of piRNA-related proteins (green) in E18.5 WT (top) and Hsp90α KO (bottom) testis. Bars: *A*: 50 μm *B*: 20 μm. (**C**) MIWI2 was immunoprecipitated from the E16.5 testes of WT or Hsp90α KO mice and subjected to western blotting. Immunoprecipitants were blotted for MIWI2 (top), methyl-arginine (middle) and WDR77 (bottom). See also Supplementary Figure S1.

Immunofluorescence analysis revealed that HSP90α is specifically expressed in germ cells (prospermatogonia) in the testis on embryonic day 16.5 (E16.5), whereas HSP90β is expressed in both somatic and germ cells, suggesting a germ cell-specific function of HSP90α (Figure 1A). The prospermatogonia produce piRNAs at this developmental stage, and a deficiency in the biogenesis of fetal piRNAs in animals with mutations in piRNA-related genes such as *Mili*, *Miwi2*, *Tdrd1*, *Tdrd9*, *Mov10l1*, *Maelstrom* and *Mitopld* leads to a failure in spermatogenesis (10). We therefore investigated whether subcellular localization and/or expression levels of piRNA-related proteins are affected by the *Hsp90α* KO mutation in E18.5 testes. Proteins involved in piRNA biogenesis co-localize at granules around the periphery of the nucleus. MILI, one of the two PIWI proteins expressed in prospermatogonia, and TDRD1 localize at cytoplasmic granules at the periphery of nucleus, called pi-bodies, whereas the other PIWI protein MIWI2 as well as TDRD9 and MAELSTROM localizes at different cytoplasmic granules around the nucleus, called piP-bodies (28). MIWI2 and TDRD9 also localize in the nucleus, which is thought to be important for piRNA-targeted DNA methylation (25,29). In *Hsp90α* KO mice, MILI expression and localization were unaffected (Figure 1B and Supplementary Figure S1A). However, we detected an obvious difference in MIWI2 localization. Although MIWI2 localizes both at perinuclear granules and in the nucleus in the WT germ cells, nuclear staining was significantly decreased in *Hsp90α* KO germ cells (Figure 1B), although some staining still remained in nuclei. At the mRNA level, the *Miwi2* expression was not affected in *Hsp90α* KO testes at E16.5 (Supplementary Figure S1B).

Because MIWI2 interacts with TDRD9, a Tudor domain-containing protein (25,30), we investigated TDRD9 expression in *Hsp90α* KO mice as well as the expression of another Tudor protein, TDRD1, which interacts with MILI. The localization of TDRD9 and TDRD1 was unchanged in *Hsp90α* KO mice (Figure 1B). The expression and localization of the other proteins involved in fetal piRNA biogenesis, MAELSTROM and MOV10L1, were also unaffected in *Hsp90α* KO mice (Supplementary Figure S1C).

### MIWI2 was methylated at arginines in WT and *Hsp90α* KO testes

The Tudor domains in various proteins specifically bind to methylated arginine residues (31), and it has been postulated that the MIWI2-TDRD9 interaction depends on the methylation of arginine residue(s) in MIWI2 at its RA/RG motifs, which is likely catalyzed by an arginine methyltransferase, PRMT5 (32,33). PRMT5 is a client protein of HSP90, and PRMT5 and HSP90α were found to interact with MIWI2 (32). Moreover, chemical inhibition of HSP90 activity severely diminishes the stability of the PRMT5 protein in cultured cells (34). Therefore, we investigated the arginine methylation of MIWI2 in WT and *Hsp90α* KO mice. For this purpose, MIWI2 was first immunoprecipitated in whole cell lysates of E16.5 testes, and the precipitants were analyzed by western blotting against an anti-methylarginine antibody. We detected an arginine-methylated protein in the WT preparation that co-migrated with MIWI2 in the gel (Figure 1C), suggesting that MIWI2 is indeed methylated at arginine(s) in prospermatogonia. The level of arginine methylation was comparable between WT and KO preparations, suggesting that the arginine methylation of MIWI2 is not disturbed in *Hsp90α* KO mice. Presumably, HSP90β may facilitate PRMT5 activity. WDR77, a co-factor of PRMT5, was detected in the MIWI2-IP fraction of *Hsp90α* KO testes, and the amount was similar to that of WT testes (Figure 1C).

Our results together suggest that in the absence of HSP90α, MIWI2 fails to enter the nucleus even though MIWI2 is methylated and its associated partners, TDRD9 and MAELSTROM, are properly expressed and localized. Failure in MIWI2 translocation is also observed in *Mili*, *Tdrd1*, *Tdrd12*, *Maelstrom*, *Mov10l1* and *Fkbp6* mutant mice (15,25,35–37), all of which are deficient in piRNA biogenesis to various degrees. Therefore, we analyzed small RNAs from WT and *Hsp90α* mutant testes. For comparison, we also analyzed published small RNA sequencing data for *Mili*, *Miwi2* and *Fkbp6* KO testes and their WT controls.

### *Hsp90α* mutation displayed a remarkable decrease in fetal piRNAs

Fetal piRNAs are classified into two types: primary and secondary piRNAs. Primary piRNAs are generated from precursor RNAs transcribed from ∼200 genomic regions called piRNA clusters. Transposon-derived RNAs are also the sources of the primary piRNAs. These primary piRNAs preferentially have uracil at the first position, and they are loaded onto MILI. MILI cleaves RNAs containing transposon sequences that are complementary to the primary piRNA. This generates secondary piRNAs, which preferentially have adenine at the 10th position. The secondary piRNAs are loaded onto either MILI or MIWI2 to guide the cleavage of complementary RNAs, a process called the ping-pong cycle. We therefore analyzed both types of piRNAs as well as miRNAs.

**Figure 2.**
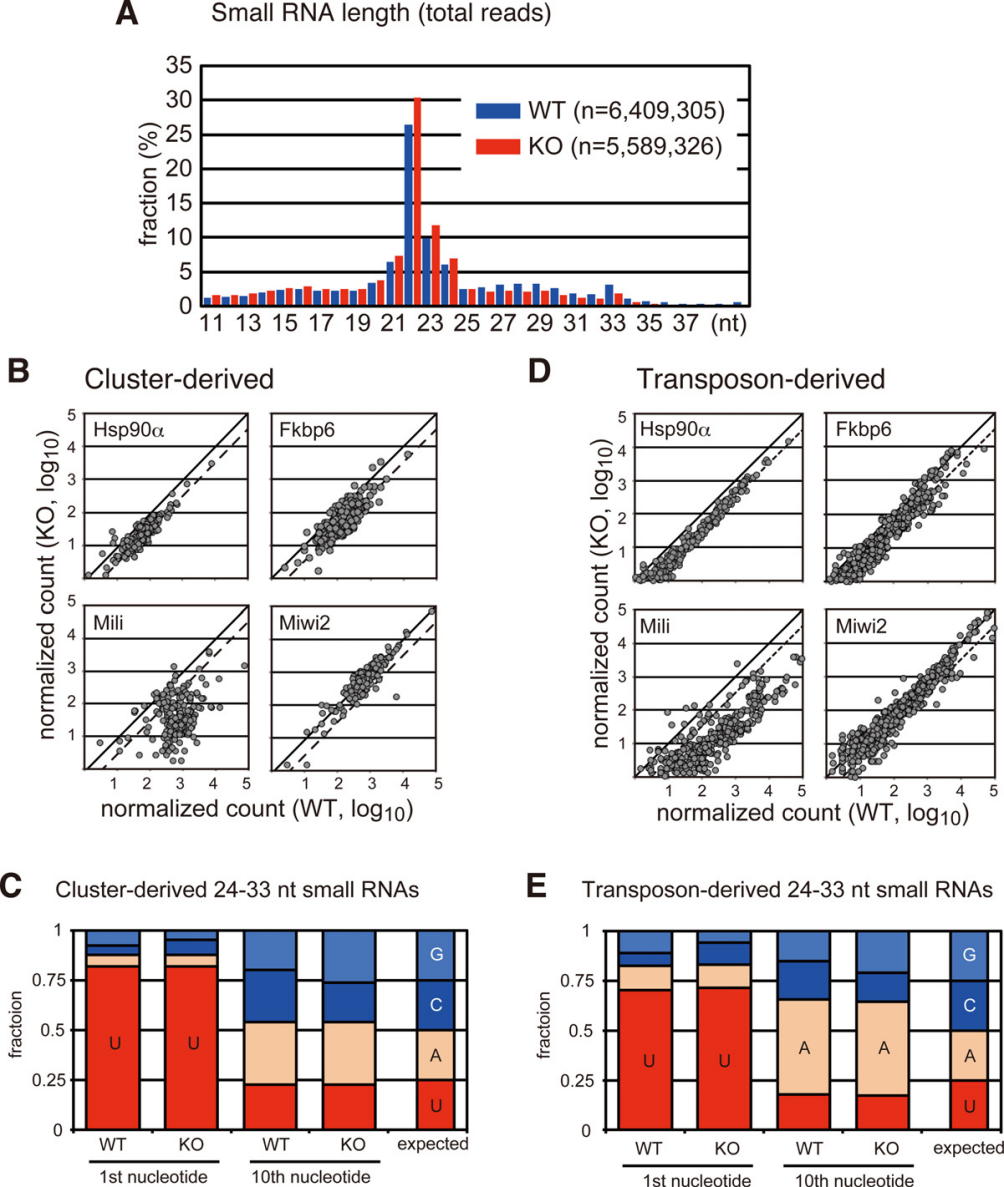
The *Hsp90α* KO mutation reduced both cluster- and transposon-derived small RNAs in E16.5 testes. (**A**) Length distributions of small RNA sequences in WT (blue) and *Hsp90α* KO (red) fetal testes. (**B**, **D**) Small RNA sequence reads (24–33 nt) from *Hsp90α*, *Fkbp6*, *Mili* and *Miwi2* KO testes (vertical axis), which were uniquely mapped to the fetal piRNA clusters (B) or the consensus sequences of mouse transposons (D), are plotted against those from the respective WT controls (horizontal axis). Each plot represents one of the clusters (B) or transposons (D). All sequence reads are normalized as *per million miRNA reads*. The dashed line indicates a 3-fold reduction in the KO library, and the solid line indicates no change. (**C**, **E**) The nucleotide bias at the 1st and 10th positions in the 24–33-nt small RNAs mapped to the piRNA clusters (C) and transposons (E) in WT and KO testes.

Small RNA libraries were generated from WT and *Hsp90α* KO testes at E16.5 and deeply sequenced (Figure 2A). The miRNA reads in the WT and KO libraries were comparable (83.2 and 83.9% of the total 20–23-nt RNAs, respectively). In contrast, the levels of 24–33-nt small RNAs uniquely mapped to the previously identified fetal piRNA clusters, representing primary piRNAs, were reduced by ∼3-fold in the KO testes (Figure 2B and C). The levels of 24–33-nt small RNAs mapped to transposon sequences, consisting of primary and secondary piRNAs, were also reduced by ∼3-fold (Figure 2D and E). Similar trends were observed in the *Fkbp6* co-chaperone mutant (Figure 2B and D; Xiol *et al.* (15)); however, the effect was larger in *Hsp90α* mutants than in *Fkbp6* mutants. The *Fkbp6* mutation reduced the expression of small RNAs in the range of 28–32 nt, the size range of MIWI2-bound piRNAs; however, it did not affect 24–27-nt RNAs, the size range of MILI-bound piRNAs (Supplementary Figure S2A). In contrast, the *Hsp90α* mutation equally suppressed the expression of 24–32-nt RNAs (Supplementary Figure S2C), suggesting the presence of both FKBP6-dependent and FKBP6-independent actions of HSP90α. In any event, these results indicate that HSP90α plays a role in the biogenesis and/or stability of piRNAs in prospermatogonia. Whereas the *Mili* mutation severely downregulates both cluster- and transposon-derived piRNAs, the *Miwi2* mutation does not reduce cluster-derived piRNAs (Figure 2B and D; Aravin *et al.* (35)). Therefore, the downregulation of both types of piRNAs in *Hsp90α* KO germ cells suggests that HSP90α affects both the activity of MIWI2 and the upstream reaction(s) in the piRNA biogenesis pathway, possibly primary piRNA biogenesis and/or stability.

**Figure 3.**
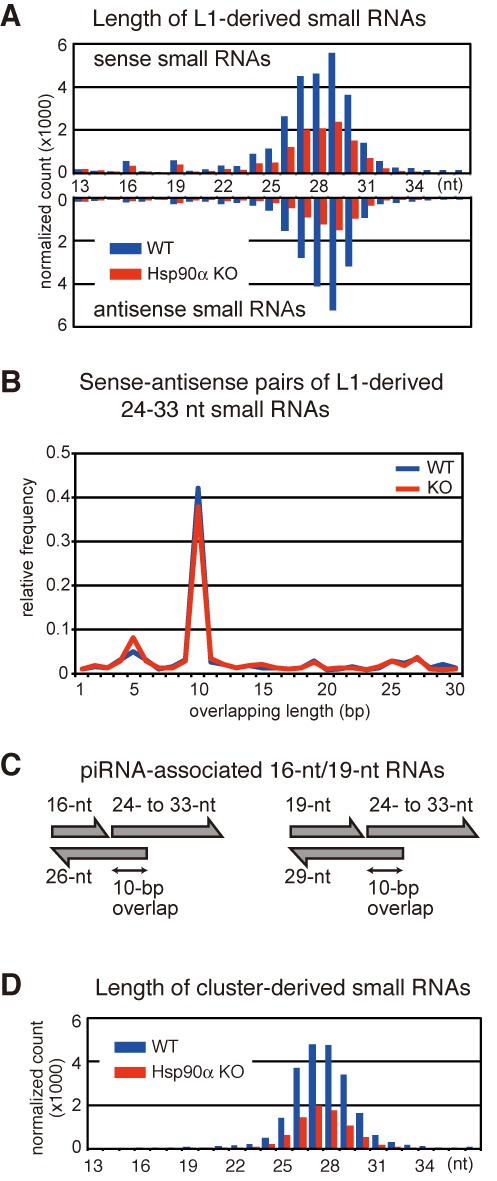
The *Hsp90α* KO mutation reduced L1-derived piRNAs, but not piRNA-related 16- or 19-nt small RNAs. (**A**) The length distributions of small RNAs in the WT (black) and *Hsp90α* KO (red) libraries that were mapped to the consensus sequence of the A-type L1 retrotransposon. The sense (top) and antisense (bottom) RNAs are separately presented. (**B**) Length distribution of distances between the 5′-end of 24–33-nt small RNAs mapped to the sense strand of the L1 sequence and the 5′-end of 24–33-nt small RNAs mapped to the L1 antisense strand. Blue line, WT library; red line, KO library. (**C**) Configuration of 16- and 19-nt small RNAs derived from the L1 sequence. For many 16- and 19-nt small RNAs, their end positions are 1 bp upstream of the start position of 24–33-nt small RNAs on the same strand, and their start positions are the same position as the end position of 26- and 29-nt small RNAs on the opposite strand. The two piRNA-length small RNAs overlap each other by 10 bp. See also Supplementary Figure S2 for details. (**D**) The length distributions of small RNAs in the WT (blue) and *Hsp90α* KO (red) libraries that were mapped to the previously identified piRNA clusters.

To study the role of HSP90α in the piRNA pathway in more detail, we analyzed small RNAs mapped to the consensus sequence of A-type LINE-1 (L1), one of the most active retrotransposons in the mouse genome (Figure 3A). Both sense and antisense small RNA levels were reduced in the KO testes. Although this global reduction, the mapped 24–33-nt small RNAs in the KO library displayed a bias toward uracil at the first position and adenine at the 10th position as well as a 10-bp overlap between sense and antisense small RNAs (Figures 2C, E and 3B), which are the hallmarks of piRNAs generated via the ping-pong cycle. We also detected an ∼3-fold reduction in the expression of piRNAs derived from Gf-type L1, another active L1 subfamily, in KO testes.

In addition to the length range of typical piRNAs, we detected a number of 19-nt small RNAs, which were positioned immediately upstream of 24–33-nt piRNAs on the same strand and were complementary to 29-nt piRNAs on the opposite strand (Figure 3A, C, and Supplementary Figure S2). Most (79%) 19-nt RNAs were associated with piRNAs that have adenine at the 10th position, and the 19-nt peak was not observed for cluster-derived small RNAs (Figure 3D). Therefore, these 19-nt RNAs are likely the byproducts of piRNA production via the ping-pong cycle, as has been suggested for 19-nt RNAs present in adult testes containing pachytene and prepachytene piRNAs (38,39). In addition, the presence of 16-nt byproducts was evident (Figure 3A, C and Supplementary Figure S2). The amounts of these 19- and 16-nt byproducts were only slightly reduced by the *Hsp90α* mutation. It has been reported that in cultured silkworm cells, chemical inhibition of HSP90 results in the accumulation of such 16-nt byproducts in the PIWI complex, suggesting that HSP90 facilitates the recycling of PIWI for efficient ping-pong cycling (15). In the mouse system, however, our data imply that neither RNA cleavage nor PIWI recycling is affected by HSP90α inactivation; thus, it appears more likely that HSP90α plays a role in stabilizing 24–33-nt piRNAs, such as stabilizing piRNA–protein complexes.

### L1 retrotransposons are derepressed in the fetal germ cells of *Hsp90α* KO mice

**Figure 4.**
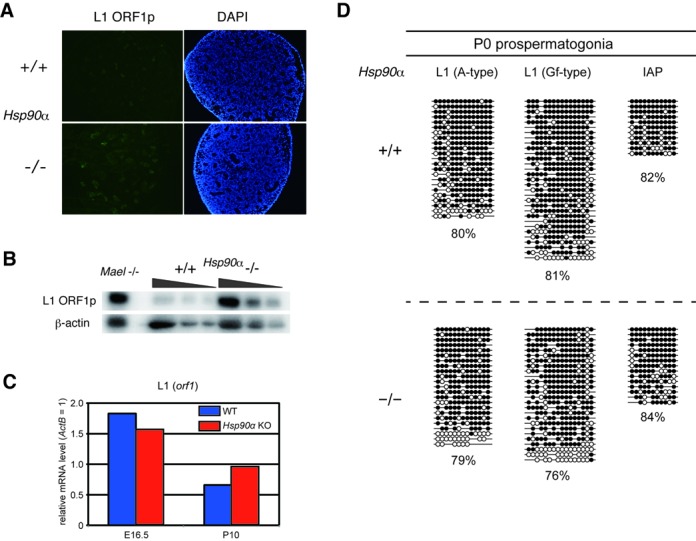
Increase in L1-encoded protein levels in *Hsp90α* KO testes. (**A**) Immunofluorescence detection of L1 ORF1 protein in E18.5 testes. (**B**) Western blot analysis of L1 ORF1 protein in E16.5 testes lysates. *Maelstrom* KO testis lysate (gifted from Dr A. Bortvin) was used as a positive control, and β-actin served as a loading control. (**C**) The gene expression of L1 *Orf1* in WT (blue) and *Hsp90α* KO (red) testes at E16.5 and P10 was determined by quantitative RT-PCR and normalized by the *ActB* expression level. (**D**) DNA methylation levels of retrotransposon sequences in WT and KO prospermatogonia (at P0) determined by bisulfite-PCR sequencing. Open and closed circles represent unmethylated and methylated CpG sites, respectively.

The reduction of L1-derived 24–33-nt piRNAs prompted us to investigate whether the expression of this retrotransposon is misregulated in mutant prospermatogonia. Thus, we compared the expression of the L1-encoded ORF1p protein in WT and *Hsp90α* KO testes at E18.5 by immunofluorescence. The number of ORF1p-positive germ cells was significantly increased in the fetal *Hsp90α* KO testis (Figure 4A). Furthermore, western blotting of the whole testes lysates (E16.5) revealed a definite increase in ORF1p levels in the mutant testes (Figure 4B). In contrast, L1 mRNA levels were largely unchanged in these testes (Figure 4C), suggesting that the effect occurs at the post-transcriptional level. Consistently, the DNA methylation levels of the promoter sequences of A- and Gf-type L1 were largely similar between WT and KO P0 prospermatogonia (Figure 4D).

### Postnatal piRNA levels derived from retrotransposons were reduced in *Hsp90α* KO mice

**Figure 5.**
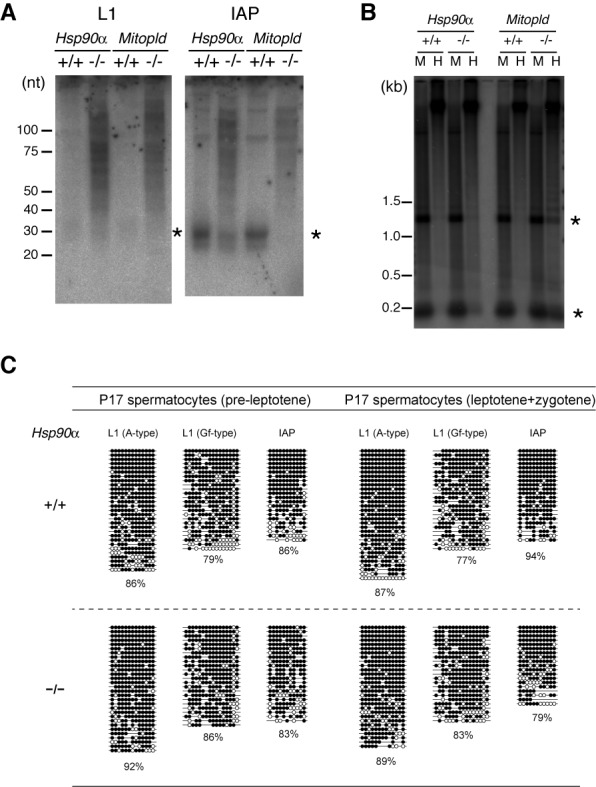
The expression levels of piRNA and the DNA methylation of L1 and IAP retrotransposons. (**A**) Northern blot analysis of piRNAs derived from L1 and IAP in P24 testes. Mitopld KO testes were used as a control for piRNA defects. (**B**) DNA methylation state of L1 5′-UTR was detected by Southern blot analysis of testes DNA digested by a methylation-insensitive (MspI; M) or methylation-sensitive (HpaII; H) restriction enzyme. (A, B) Asterisks indicate the expected band sizes. (**C**) DNA methylation levels of retrotransposon sequences in WT and KO spermatocytes (at P17) determined by bisulfite-PCR sequencing. Open and closed circles represent unmethylated and methylated CpG sites, respectively.

We also investigated the effect of the *Hsp90α* KO mutation on piRNA biogenesis and DNA methylation in postnatal germ cells. Northern blot analysis of testis RNA on postnatal day 24 (P24) against piRNA derived from IAP, another active retrotransposon, revealed a severe reduction in piRNA levels in the mutant testes (Figure 5A). The reduction of L1-derived piRNA was unclear because of smearing; however, longer RNAs were accumulated in the mutant testes, very similarly to *Mitopld* KO testes, which are deficient in piRNA production and show an increased L1 mRNA level at postnatal stages (18). These results suggest that, at the postnatal stages, L1 transcription is increased in *Hsp90α* KO testes. The DNA methylation level in the L1 promoters was partially reduced in the *Hsp90α* mutant testes at P15 when analyzed by methylation-sensitive enzyme digestion and Southern blot, although it was not as much as *Mitopld* KO testes (Figure 5B). However, when analyzed by bisulfite sequencing, the L1 promoters were normally methylated in *Hsp90α* KO meiotic germ cells (spermatocytes) at P17 (Figure 5C), as in the P0 prospermatogonia (Figure 4D). Although there might be a difference in methylation in several limited loci of L1, the profound decrease in piRNA production did not affect DNA methylation of a bulk of L1 promoters.

## DISCUSSION

The HSP90 proteins play a pivotal role for maintaining the integrity of cellular functions by supporting the activities of many client proteins, and this HSP90's function has been proposed to be important for the robustness of developmental pathways under genetic or environmental perturbations (i.e. canalization) (40,41). In the germ cells, maintaining the integrity of the genome and epigenome is especially important, because they convey the genetic and possibly epigenetic information to the progenies. The germline piRNA system is an efficient host defense system against the mutagenic effects of active transposons by cleaving their RNAs and epigenetically silencing their promoters. We recently revealed that Hsp90α is essential for spermatogenesis (17), and in this report, we showed that HSP90α is important for piRNA production and post-transcriptional repression of retrotransposons in mouse fetal germ cells. Several proteins indispensable for piRNA production have been identified, and there are even more proteins regulating these key proteins. The PIWI-clade Argonaute proteins, MILI and MIWI2, are the most centered in the mouse fetal piRNA system as they form complexes with primary piRNAs, cleave the transposon-derived RNAs to generate secondary piRNAs and mediate DNA methylation of transposons such as L1. The loss of HSP90α decreased both MILI- and MIWI2-bound fractions of fetal piRNAs by ∼3-fold, but not completely. The remaining piRNAs showed the hallmarks of ping-pong cycle, which indicate that the RNase (slicer) activity of the PIWI protein(s) is still working in KO germ cells. Nevertheless, the decrease was enough to derepress retrotransposons.

The reports on the piRNA system have been accumulating in this decade. However, the mechanisms of piRNA-mediated retrotransposon silencing are still not fully understood, especially in the series of steps: (i) the loading of MILI-generated piRNAs onto MIWI2, (ii) the translocation of MIWI2–piRNA complex into nucleus and (iii) the introduction of DNA methylation in retrotransposon promoters. TDRD9, which contains a Tudor domain recognizing methylated arginine residues, has been shown to co-localize with and binds to MIWI2 at piP-bodies and both proteins are also present in nucleus, suggesting its role in the MIWI2 translocation (25). The other PIWI proteins in postnatal germ cells, MILI and MIWI, have been shown to be methylated by an HSP90 client protein, PRMT5, which is a type II protein methyltransferase (32,33). However, it was unknown whether MIWI2 is methylated *in vivo*, although MIWI2 has the PRMT5-methylation motif and can be methylated by PRMT5 *in vitro* (32,33). Here, we showed that MIWI2 is indeed methylated in fetal germ cells. But, it is likely that the level of the MIWI2 methylation was not affected in prospermatogonia at E16.5 by the *Hsp90α* deficiency, demonstrating that the arginine methylation is not sufficient for the MIWI2 protein to translocate into nuclei. Interestingly, even with the arginine methylation of MIWI2, the subcellular localization patterns of MIWI2 and TDRD9 became different in *Hsp90α* KO germ cells; TDRD9 still can enter the nucleus in the mutant. Given the function of HSP90α in protein transport across membranes in dendritic cells (16,24), it is tempting to speculate that HSP90α may function as a chaperone for MIWI2 translocation. This prospect should be explored in future studies.

Despite the low efficiency of MIWI2 nuclear translocation and the low amount of piRNAs, the DNA methylation levels at L1 promoters were not severely affected. Therefore, the small amounts of MIWI2–piRNA complexes in the nucleus might be enough to guide *de novo* DNA methylation of the retrotransposon promoters. We showed that L1 mRNA is highly produced even in the WT fetal testes and is not extra-elevated in the *Hsp90α* KO testes. On the other hand, the amount of the L1-encoded protein was significantly elevated in the *Hsp90α* mutant testes. These features, i.e. *de novo* DNA methylation is not affected but the expression of L1 is elevated at the protein level, are very similar to the phenotype of the mutant of *Maelstrom* (28), a gene known to be involved in the piRNA-based transposon silencing system. Therefore, L1 is repressed at the post-transcriptional level in fetal male germ cells (prospermatogonia), and Maelstrom and HSP90α are likely involved in it. On the other hand, the piRNA-guided DNA methylation at the L1 promoter is important for transcriptional silencing in later (postnatal) stages, as evidenced by an increase in L1 mRNA after birth in the *Mili* and *Miwi2* mutants (23,42–43). HSP90α is possibly involved in several distinct steps in normal male germ cell development, because HSP90 is a chaperone that stabilizes a numerous client proteins in cytosol and in nucleus. Further study is needed to elucidate whether the deficiency in post-transcriptional regulation of retrotransposons in the *Hsp90α* KO fetal germ cells is directly linked to the deficiency in piRNA biogenesis.

Involvement of the HSP90 chaperone complexes in the mouse piRNA biogenesis was first suggested in the study of FKBP6, HSP90's co-chaperone, in which the authors showed that the *Fkbp6* KO mice had a defect in piRNA production, especially in MIWI2-bound piRNA (15). They also analyzed the effect of an HSP90 inhibitor, Geldanamycin (GA), on the piRNA biogenesis in cultured silkworm cells. The GA-treatment resulted in the accumulation of additional ∼16-bp small RNA species which are likely byproducts of the RNA cleavage reaction by Ago3, a PIWI protein. Thus, they suggested that FKBP6 in mice and HSP90 in silkworm are involved in efficient recycling of the slicer enzyme. On the other hand, our computational analysis on the published sequence data revealed that the ping-pong byproducts are not accumulated in *Fkbp6* KO testes, similarly to the *HSP90α* KO testes. It is of note that, in *Hsp90α* KO testes, not only MIWI2-bound piRNAs but also MILI-bound piRNAs are reduced, indicating the presence of both FKBP6-dependent and FKBP6-independent actions of HSP90α. This seems to parallel with the insect piRNA system where at least two different HSP90 co-chaperones, Hop and Shutdown (Fkbp6 ortholog), are required (12,14). The reduction of both MILI- and MIWI2-bound piRNAs by the *Hsp90α* mutation also suggests that the steps where HSP90α is involved in the piRNA biogenesis pathway include a step(s) upstream of the enzymatic reactions by MILI. Therefore, in analogy to the role of the insect HSP90 machinery in the formation of piRNA–PIWI complex (13) and siRNA–Ago2 complex (8), it is conceivable that the mouse HSP90α machinery has important roles in the formation and/or stability of the MILI–piRNA and MIWI2–piRNA complexes, the later being dependent on the FKBP6 co-chaperone.

The two cytosolic HSP90 isoforms were generated by gene duplication in the common ancestor of vertebrates and maintained thereafter, with the amino acid sequence and the expression pattern being diverged (44). However, whether the two isoforms are *functionally* diverged, particularly whether HSP90α with more tissue-specific expression has indispensable functions, remains controversial. A function specific to HSP90α would be its extracellular role in regulating matrix metalloproteinase activity and cancer metastasis (45). However, little is known about the difference in their intracellular functions. We previously demonstrated that HSP90α plays a more important role than HSP90β in the membrane translocation and presentation of exogenous antigens in mouse dendritic cells (16,24). In this study, our data revealed that HSP90α plays specific roles in the epigenetic regulatory systems against transposons during germ cell development. The single insect gene of HSP90 is also involved in piRNA biogenesis and transposon silencing (11,12), hinting that the function of the HSP90 machinery in piRNA biogenesis is conserved in metazoans. After gene duplication and functional divergence, this piRNA-related function may have become specific to *Hsp90α* in vertebrates or at least in mammals. Then, under the selection, *Hsp90α* may have become highly expressed in male germ cells.

## ACCESSION NUMBERS

The small RNA sequencing data were deposited in GEO under the accession no. GSE54515.

## SUPPLEMENTARY DATA

Supplementary Data are available at NAR Online.

SUPPLEMENTARY DATA
