# Supplementary material for: HSP90α plays an important role in piRNA biogenesis and retrotransposon repression in mouse
Source: Nucleic Acids Res. 2014 Sep 27;42(19):11903–11. doi: 10.1093/nar/gku881 (PMC4231750; doi:10.1093/nar/gku881)
Supplement: SUPPLEMENTARY DATA [file supp_42_19_11903__index.html]

HSP90α plays an important role in piRNA biogenesis and retrotransposon repression in mouse — HSP90α plays an important role in piRNA biogenesis and retrotransposon repression in mouse — SUPPLEMENTARY DATA 

# HSP90α plays an important role in piRNA biogenesis and retrotransposon repression in mouse

## SUPPLEMENTARY DATA

**Files in this Data Supplement:**

- SUPPLEMENTARY DATA
