# Supplementary material for: HSP90α plays an important role in piRNA biogenesis and retrotransposon repression in mouse
Source: Nucleic Acids Res. 2014 Sep 27;42(19):11903–11. doi: 10.1093/nar/gku881 (PMC4231750; doi:10.1093/nar/gku881)
Supplement: SUPPLEMENTARY DATA [file supp_gku881_nar-01830-x-2014-File007.pdf]

Ichiyanagi, T et al.

Supplemental Figures

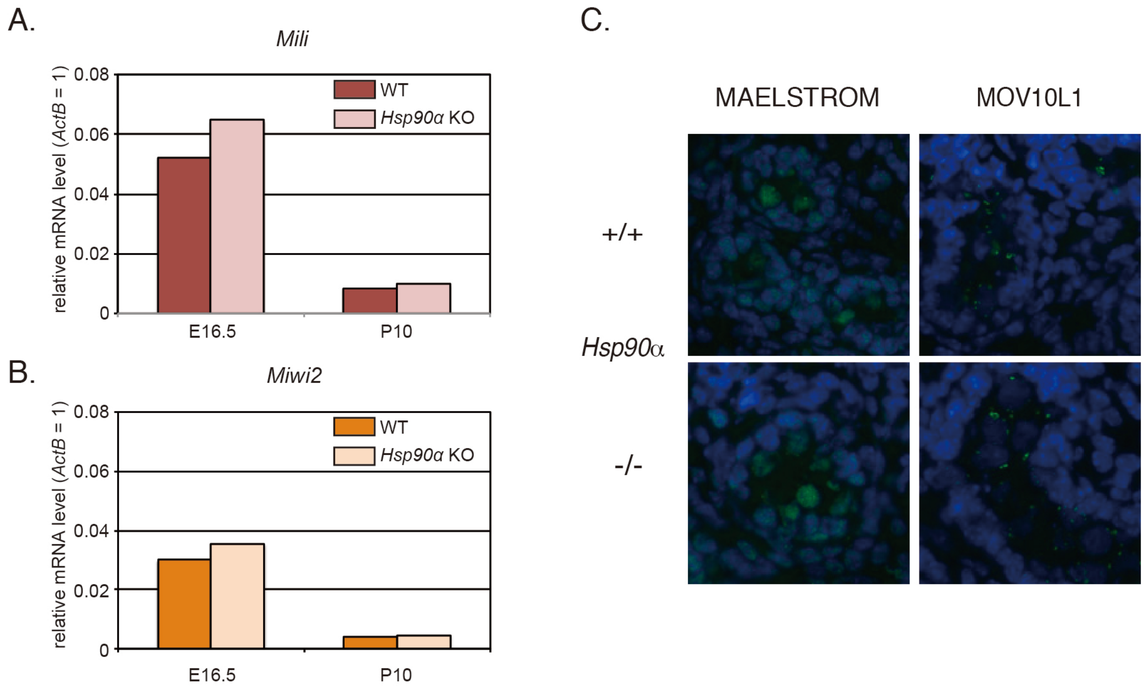

Figure S1 piRNA-related gene expressions and protein localizations.

(A, B) The gene expression of *Mili* (A) and *Miwi2* (B) in WT (dark colors) and *Hsp90α* KO (light colors) testes at E16.5 and P10 was determined by quantitative RT-PCR. All data were normalized by the *ActB* expression level. We did not detect large differences in *Mili* and *Miwi2* expression between WT and KO testes. (C) Localization of MAELSTROM (green; *left*) and MOV10L1 (green; *right*) was determined by immunofluorescence staining of E18.5 testis frozen sections. Nuclei were counter-stained with DAPI (blue).

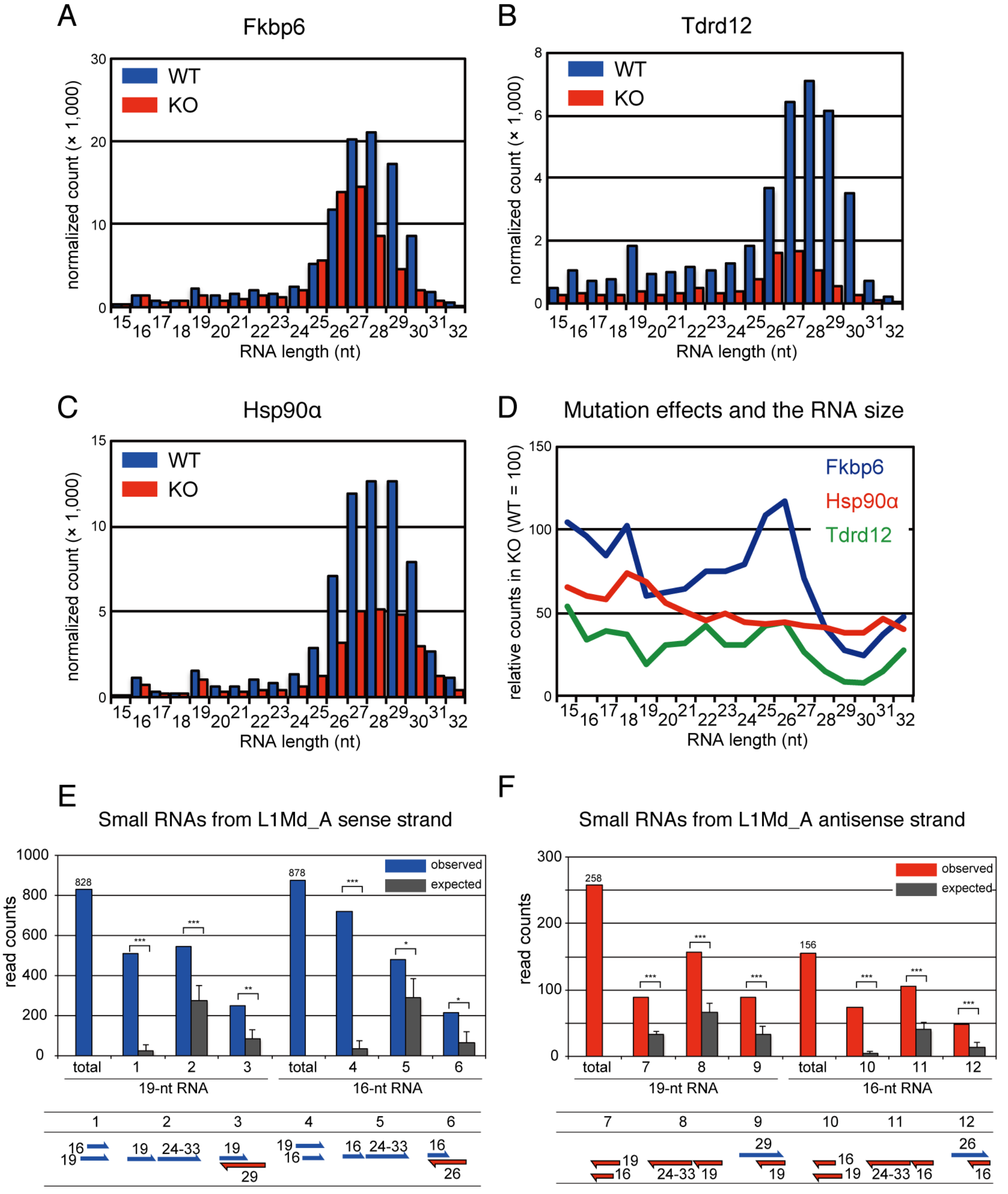

Figure S2 Length distributions of transposon-derived small RNAs and configuration of 16- and 19-nt small RNAs.

(A, B, C) Small RNAs in *Fkbp6*, *Tdrd12*, and *Hsp90α* KO libraries (red) and their WT controls (blue) were mapped to transposon consensus sequences, and mapped reads of indicated lengths were counted. The read counts were normalized by miRNA levels in the respective libraries as *reads per million miRNA reads*. (D) The reduction by the KO mutations was calculated for RNAs of the indicated lengths.

The *Fkbp6* and *Tdrd12* mutants exhibited a severe reduction in piRNAs of 28 nt or longer (typically MIWI2-bound), whereas the *Hsp90a* mutants displayed a reduction in piRNAs of 24–27 bp (typically

MILI-bound), in addition to piRNAs of 28 nt or longer. In the *Tdrd12* mutant, MIWI2 does not bind small RNAs, and the slicer activity of MILI is proposed to be affected (Pandey *et al.* 2013, Proc. Natl. Acad. Sci. USA 110:16492-16497). Our analysis revealed that 16- and 19-nt RNAs were diminished in the *Tdrd12* mutant, which is consistent with the inference that these 16- and 19-nt RNAs are byproducts of the slicing reactions to generate secondary piRNAs.

(E, F) Mapping positions of 16- and 19-nt RNAs derived from sense (E) and antisense (F) strands of the L1 retrotransposon were compared with those of other small RNAs. Configuration of 16- and 19-nt small RNAs suggests they are byproducts of RNA cleavage during the ping-pong cycle. The blue and red bars denote the exact read counts for the indicated RNA in the indicated configurations. (1) Sense-strand 19-nt RNAs whose 3′-ends are at the same position as the 3′-end of at least one sense-strand 16-nt RNA. (2) Sense-strand 19-nt RNAs whose 3′-ends are 1 bp upstream of the 5′-end of at least one sense-strand 24–33-nt RNA. (3) Sense-strand 19-nt RNAs whose 5′-ends are at the same position as the 3′-end of at least one antisense-strand 29-nt RNA. (4) Sense-strand 16-nt RNAs whose 3′-ends are at the same position as the 3′-end of at least one sense-strand 19-nt RNA. (5) Sense-strand 16-nt RNAs whose 3′-ends are 1 bp upstream of the 5′-end of at least one sense-strand 24–33-nt RNA. (6) Sense-strand 16-nt RNAs whose 5′-ends are at the same position as the 3′-end of at least one antisense-strand 26-nt RNA. (7) Antisense-strand 19-nt RNAs whose 3′-ends are at the same position as the 3′-end of at least one antisense-strand 16-nt RNA. (8) Antisense-strand 19-nt RNAs whose 3′-ends are 1 bp upstream of the 5′-end of at least one antisense-strand 24–33-nt RNA. (9) Antisense-strand 19-nt RNAs whose 5′-ends are at the same position as the 3′-end of at least one sense-strand 29-nt RNA. (10) Antisense-strand 16-nt RNAs whose 3′-ends are at the same position as the 3′-end of at least one antisense-strand 19-nt RNA. (11) Antisense-strand 16-nt RNAs whose 3′-ends are 1 bp upstream of the 5′-end of at least one antisense-strand 24–33-nt RNA. (12) Antisense-strand 16-nt RNAs whose 5′-ends are at the same position as the 3′-end of at least one sense-strand 26-nt RNA. If a sense RNA satisfies (2) and (3), then the sense 24–33-nt RNA and antisense 29-nt RNA are involved in the ping-pong cycle, as shown in Fig. 3E. Expected values (gray) were the averages calculated from 1000 simulated datasets in which the mapping positions of 16- or 19-nt RNAs were randomized. Error bars indicate standard deviations. The p-values were calculated from the aforementioned simulations, and statistical significance is indicated by asterisks (*** $p < 0.001$, ** $p < 0.01$, * $p < 0.05$).

Supplemental Table

Table S1. Oligonucleotides used in this study

	name	forward primer	reverse primer	ref.
RT-qPCR	L1_ORF1	GGTGAGGAAATGAACAAAACC	ATGCTCGCATCTATGGTTCC	this study
RT-qPCR	L1_5'UTR	GGCGAAAGGCAAACGTAAGA	GGAGTGCTGCGTTCTGATGA	Watanabe et al. Dev.Cell 2011, Carmell et al.Dev.Cell 2007
RT-qPCR	Miwi2	ATTTGACGGTGCCAGCCTTT	ACAGGGGAGTTTGGGAAGAG	this study
RT-qPCR	Mili	ATAAGCAGGGAACGATGCTG	CTTGTTGAGGGCATCTCTGC	this study
RT-qPCR	ActB	CCAACCGTGAAAAGATGACC	CCATCACAATGCCTGTGGTA	this study
Southern	L1Md_A2_ORF	TCCCAACATAGAGTCCTGAG	AGTGGGCAGAGTATTCTCTG	Bourc'his and Bester, Nature 2004, Shoji et al. Dev.Cell 2009
Northern	*Line-1* piR-1831	GGGAGAAAGTGGGCAGCCTTGTCTAGTCC		Shoji et al. Dev.Cell 2009
Northern	*IAP* piR-4868	GCGCTGACATCCTGTGTTCTAAGTGGTA		Shoji et al. Dev.Cell 2009
